# Drag-Based ‘Hovering’ in Ducks: The Hydrodynamics and Energetic Cost of Bottom Feeding

**DOI:** 10.1371/journal.pone.0012565

**Published:** 2010-09-07

**Authors:** Gal Ribak, John G. Swallow, David R. Jones

**Affiliations:** 1 Department of Biology, University of South Dakota, Vermillion, South Dakota, United States of America; 2 Zoology Animal Care, University of British Columbia, Vancouver, Canada; Institut Pluridisciplinaire Hubert Curien, France

## Abstract

Diving ducks use their webbed feet to provide the propulsive force that moves them underwater. To hold position near the bottom while feeding, ducks paddle constantly to resist the buoyant force of the body. Using video sequences from two orthogonal cameras we reconstructed the 3-dimensional motion of the feet through water and estimated the forces involved with a quasi-steady blade-element model. We found that during station holding, near the bottom, ducks use drag based propulsion with the webbed area of the foot moving perpendicular to the trajectory of the foot. The body was pitched at 76±3.47° below the horizon and the propulsive force was directed 26±1.9° ventral to the body so that 98% of the propulsive force in the sagittal plane of the duck worked to oppose buoyancy. The mechanical work done by moving both feet through a paddling cycle was 1.1±0.2 J which was equivalent to an energy expenditure of 3.7±0.5 W to hold position while feeding at 1.5 m depth. We conclude that in shallow water the high energetic cost of feeding in ducks is due to the need to paddle constantly against buoyancy even after reaching the bottom. The mechanical energy spent on holding position near the bottom, while feeding, is approximately 2 fold higher than previous estimates that were made for similar bottom depths but based on the presumed motion of the body instead of motion of the feet.

## Introduction

Many diving ducks are bottom feeders that make shallow vertical dives from the surface to the benthos. They propel their body underwater by synchronized paddling with both feet while the wings are folded next to the body (some sea-duck species, e.g. eiders and scoters, use both wings and feet to descend through the water column but once at the bottom they use feet alone for propulsion [1]–[3]). Foraging on small invertebrates and sessile food does not require the diver to be particularly fast or agile underwater, and in fact, overcoming buoyancy presents the major mechanical challenge for ducks during shallow submergence. Large air volumes trapped in the waterproofed plumage, air in the air-sac system and light skeletons may be useful adaptations for floating on the water surface but these adaptations translate into high buoyancy that is energetically costly underwater [4]–[5]. Because the large air volumes in the plumage and air-sacs are compressible, the buoyancy of a duck is decreased by the increase in ambient pressure as the bird dives deeper. However, many diving ducks forage at shallow depths (<5 m) where buoyancy is still high. When descending from the surface to the bottom or while holding a vertical position near the bottom, they paddle continuously against their buoyancy. As soon as paddling stops they rise passively to the surface [6].

Wilson et al. showed that diving birds have less air in the plumage and air-sac system and higher body density than surface feeding birds [4]. Within ducks, however, Lovvorn and Jones found no difference between the buoyancy (relative to body mass) of surface feeding and diving ducks [7]. This suggests that rather than having specific adaptations for reduced buoyancy to ease diving, diving-ducks actively work against buoyancy which elevates energetic costs. Lovvorn et al. [8] and Stephenson [9] used similar biomechanical models of unsteady (acceleratory) swimming to estimate the mechanical work to reach the bottom and stay there in diving ducks. These models were based on the mechanical work done on the body, calculated from its motion (speed and acceleration, but assuming no body motion at the bottom) and suggested that diving ducks invest 36–87% of the total mechanical work of the dive as work against buoyancy. At the time, these studies highlighted the benefit of using biomechanical models as tools to break down the total energetic cost of avian diving to work done against specific forces. In this study, we continue an exploration of mechanical energy expenditure by diving ducks by focusing on the kinematics and mechanical work done directly by the feet and how these relate to the propulsive forces that keep the duck in place while feeding at the bottom.

Most, if not all, of the propulsive force is generated during a fraction of the paddling cycle, the power phase, when the webbed area of the foot is swept backwards through the water. Aigeldinger and Fish, [10] described the paddling motion of surface swimming ducklings in detail. The foot is plantarflexed and the digits abducted during the power phase while it is dorsiflexed and the webbed area collapsed during the recovery phase [10]. The power phase is typically longer than the recovery phase and occupies 66–70% of total cycle duration [8]. Underwater paddling frequency seems to increase with elevated power output [9], [11]. Paddling frequency of the ducks during descent to the bottom is 25% higher than when they remain near the bottom during feeding [9]. Ducks also decreased their paddling frequency when their buoyancy was artificially reduced by adding mass to the peritoneal cavity to increase body density [11].

Foot-propulsion in ducks has been considered as drag-based swimming because the webbed feet move backwards relative to the body in the opposite direction of forward locomotion. In contrast, wing propelled birds (e.g. alcids and penguins) utilize lift from the wings for forward thrust, and this is considered to be a more energetically efficient form of propulsion [12]–[13]. However, Johansson and Nordberg [14] showed that when the forward speed of a foot propelled bird is high, the speed of the body interacts with the motion of the foot relative to the body so that the foot is moved through the water at more moderate angles of attack. Thus, during forward swimming “drag-based” foot-propulsion actually utilizes hydrodynamic lift as the major source of forward thrust.

Detailed kinematic data on avian foot propulsion is extremely sparse. Some data is available for paddling frequency and its alteration with power requirement in ducks [8]–[9], [11]. A few studies on ducks also reported the amplitude (arc length) of the power stroke during swimming [10], [15]–[16]. Johansson and Norberg [17] provided detailed three dimensional kinematic data on the lobate toes of a Crested grebe while two dimensional data on the kinematics of the foot is also available for cormorants [14], [18]. The data on foot kinematics of the grebe and cormorant however, only refer to propulsion during horizontal swimming. The kinematics of foot propulsion during holding position near the bottom has, to the best of our knowledge, not been studied previously.

In this study we use detailed videography to analyze station holding in bottom feeding ducks. We seek to answer questions from both ecophysiological and biomechanical perspectives: 1) From an eco-physiological prospective, ducks spend most of their underwater-time feeding at the bottom. Hence, the energetic cost of feeding behavior is important to energetic models aiming to find the gain per unit effort of foraging in ducks. Thus, the ecophysiological question that underlies this study is *can we measure directly the energy expenditure of ducks holding position near the bottom*? 2) From a comparative bio-mechanical perspective, little is known about the hydrodynamic mechanism of avian foot propulsion. If fast horizontal swimming gives rise to lift based swimming in grebes and cormorants, which are specialized pursuit divers feeding on fish, *what mechanism underlies propulsion of ducks that are specialized for vertical diving and feeding at the bottom on sessile food*?

To answer these questions we extracted 3-dimensional kinematic data from movies showing the feet of diving Barrow's goldeneye ducks (*Bucephala islandica*, Gemelin, 1789) as they fed inside a dive tank. We used the data to estimate the hydrodynamic forces and work produced by the moving feet using a quasi-steady flow model.

## Materials and Methods

The ducks used in this study where kept in the Zoology Animal Care facility of the University of British Columbia. All animals were handled in strict accordance with good animal practice as defined by the relevant national and/or local animal welfare bodies, and all animal work was approved by the appropriate committee (UBC ACC#A060292)

### Modeling the propulsive force

**Figure 1:**
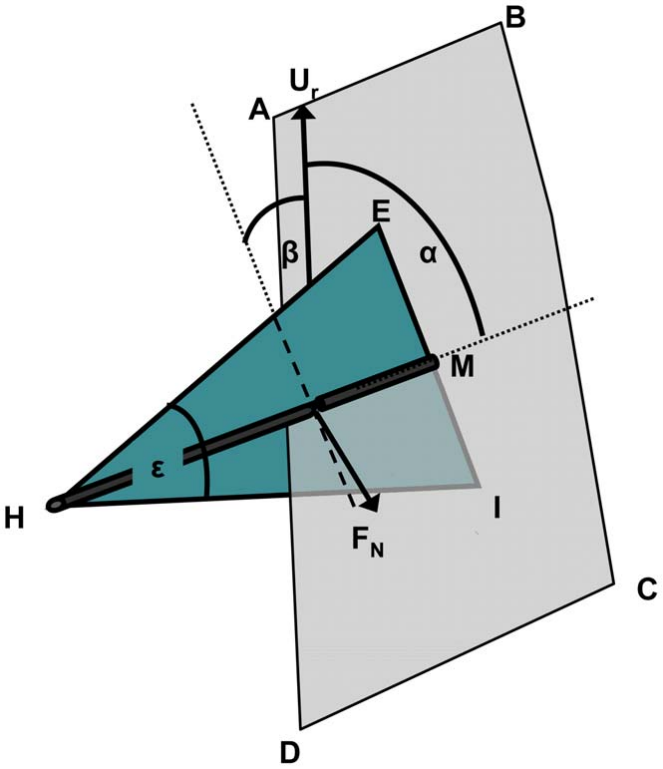
Schematic representation of the 3D geometry and notations used. The blue triangle (HIE) represents the left foot of the duck. The span of the foot is drawn as a thickened grey line (HM) that connects the base of the foot (H) with the tip of the mid digit (M). Grey area ABCD is the plane containing the span of the foot and the vector of velocity relative to water. Angles α and β are the angles between the velocity vector and the area of the foot (see text for further definitions). F_N_ is hydrodynamic force normal to the foot that results from motion of the foot through water.

Hydrodynamic forces were estimated from the 3-dimensional motion of the webbed foot through water using a variant of the “blade–element” model. Studies of fish propulsion by pectoral fins [19], frog swimming [20] and insect hovering [21] are only a few examples where the blade-element model was applied previously to study swimming and flying in animals. We describe here only the basic principles of this modeling approach whereas a detailed description of the model, as applied by us for the specific case of ‘hovering ducks’, appears in Appendix S1. The blade-element approach is based on dividing the foot into smaller elements and calculating the drag and acceleration reaction forces for each element separately. To determine the propulsive force spent holding vertical position near the bottom the model uses four kinematic parameters for each foot element over the duration of the paddling cycle. The parameters are the velocity of the foot element relative to water (***U_r_***), the acceleration of the foot element relative to water (***a_r_***) and two angles α and β that represent the orientation of the foot element relative to the flow (Fig. 1). These parameters were obtained from video sequences showing the feet of the ducks while feeding underwater.

### Apparatus

**Figure 2:**
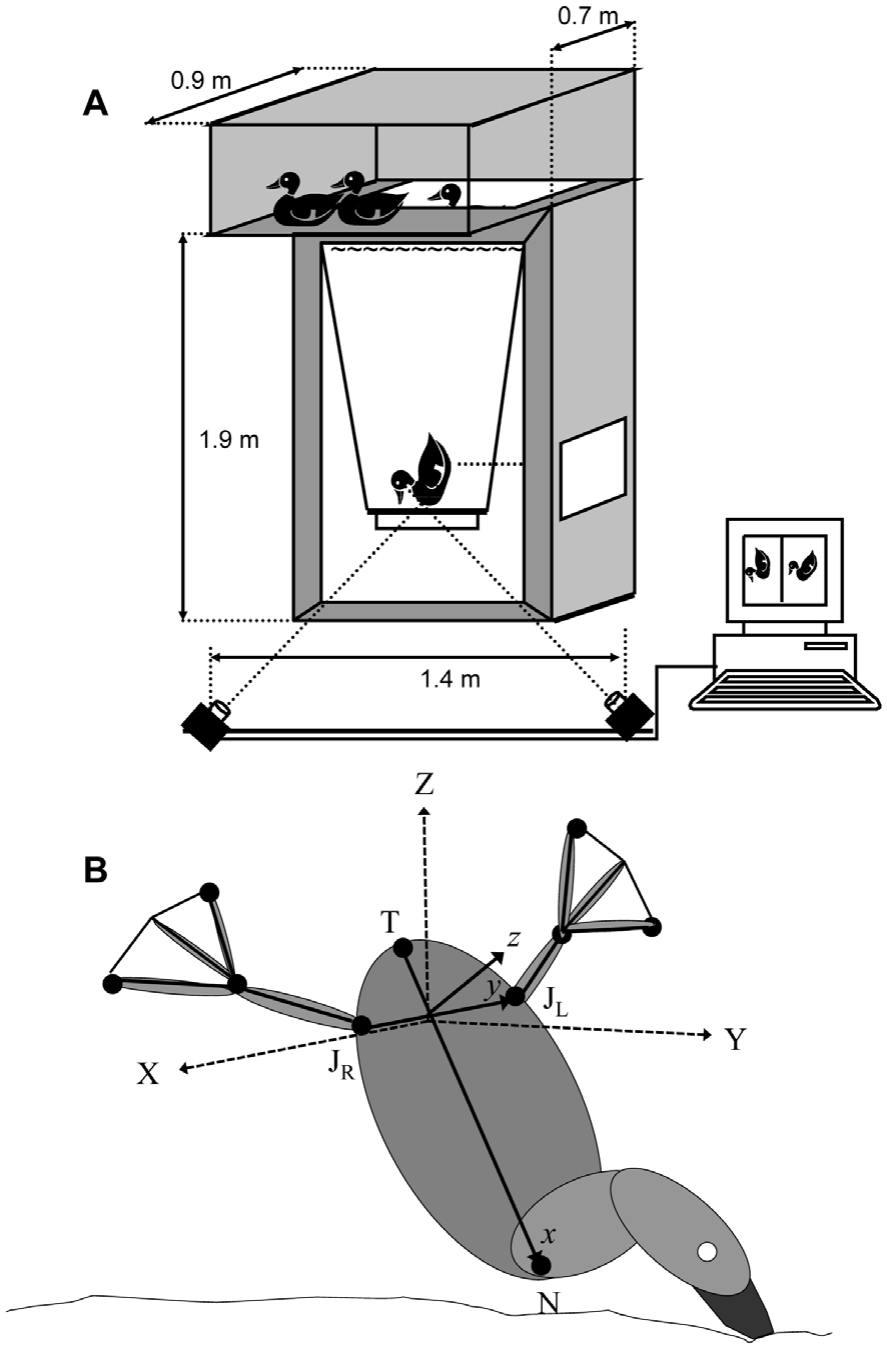
Frame of reference for the cameras and the ducks in the movies. A) The dimensions of the dive tank and camera locations. B) The coordinate system of the movies (dashed axes and upper case letters XYZ) and the transformed coordinate system that is fixed on the duck (lower case letters, xyz).

Two male and two female Barrow's goldeneye ducks (*Bucephala islandica*, Gemelin, 1789) were housed in a 1.2×0.9×0.5 m aviary mounted on top of a 1.9 m high, rectangular (1.2×0.7 m) dive tank filled with fresh water. The front wall of the tank (1.9×1.2 m) was made of transparent Perspex (Fig. 2A). The ducks were allowed free access to the dive tank and were trained to dive for grains mixed with fine pebbles (for grit) in a feeding tray that was suspended below the water surface. By gradually increasing the depth of the feeding tray the ducks were trained to perform feeding dives to 1.5 m. While feeding at the tray the ducks held their vertical position in the water column by paddling constantly. Two CCD analog cameras simultaneously filmed the ducks. The cameras were 1.4 m apart from each other, mounted on a horizontal rod parallel to the window of the tank. Each camera was oriented at 45° to the rod so that the fields of view of the orthogonal cameras intersected inside the tank. A rectangular cube with 0.4 m side dimensions was placed in the field of view of both cameras, above the feeding tray, where the ducks held position to feed. The cube was used to verify the absence of image distortion in either camera view (no curving of the sides of the cube) and for spatial calibration of the cameras. The corners on the sides of the cube were digitized and used for calculating direct linear transformation coefficients for reconstruction of 3-dimensional positions from both cameras [22]. Both cameras were connected to a desktop computer with software to control image acquisition (XCAP™, Epix, USA). The software allowed simultaneous triggering of both cameras to capture video fields (60 fields s^−1^ from each camera) for a period of 3.0 seconds.

While feeding the ducks were never completely stationary so in our analysis we chose sequences from each bird where the left foot was visible to both cameras and the position of the bird was as stable as possible. i.e., the birds had the least horizontal motion, had no turning (yaw or roll) and had a zero net vertical displacement for the paddling cycle. In each sequence we analyzed 5 consecutive paddling cycles by the same duck.

The sequences were analyzed frame by frame and 7 points in the view of each camera were digitized. Five of these points were on the legs: the tips of digits 1 and 3 (E and I in Fig. 1), the apex (H) of the webbed area of the left foot, the joint connecting the metatarsus with the tibia on the left leg (J_L_) and the same joint on the right leg (J_R_, Fig. 2B). Two additional points were on the body (Fig. 2B): the tip of the tail (T) and the connection between the neck and body (N)

### Kinematic analysis

The process of digitizing points on two spatially calibrated camera views resulted in a data set of 3D instantaneous positions for the 7 points in the earth (cameras) coordinate system (denoted by upper case letters) where Z was defined the vertical axis (with the positive direction pointing towards the surface) and X and Y were horizontal (Fig. 2). In the movies the ducks were oriented at various angles in respect to the X and Y axes (Fig. 2). To extract foot kinematics from these movies in a manner that allowed data between the different birds to be compared, data from the (X, Y, Z) frame of reference of the dive tank was transformed to a frame of reference that was fixed on the duck (denoted by lower case letters: x, y, z). We defined the origin of the xyz coordinate on the long axis of the duck (TN), as half way between J_L_ and J_R_ (Fig. 2B).

The change (with time) in position of the origin of the xyz system in XYZ coordinates was taken as the velocity of the duck (**U_b_**). The line connecting J_L_ and J_R_ defined the lateral (y) axis of the duck with the positive side pointing to the left of the duck (Fig. 2). The x and z axes were the long (positive towards the neck) and dorso-ventral (positive towards the dorsal side) axes, perpendicular to y. We used the directional cosines of these axes to give a transformational matrix transforming the positions of the 7 points and **U_b_** to the duck (x,y,z) coordinate system (see Appendix S2).

Next, we replaced the time variable within each cycle with a non-dimensional index obtained by dividing time within the cycle by the paddling cycle duration. Thus instead of actual time we had a fraction of the paddling cycle duration that was between 0 and 1. We defined the start of each paddling cycle (t_0_) when the foot started to move forward and down (recovery phase) and the cycle end (t_1_) at the end of the propulsive stroke when the foot reached its highest position. The data from each cycle was then interpolated using cubic-spline to an equally spaced abscissa ranging from 0 to 1 at intervals of 5% of paddling cycle duration. By normalizing the cycles we were able to average data from several paddling sequences even when the cycles differed in duration. Data from all sequences by the same bird (5 cycles) were averaged and we used the means of all birds. Thus in the results section we report the mean ± SD of a sample size of 4 birds.

## Results

While holding position near the feeding tray the ducks were pitched with their head pointing down and tail pointing up. The mean pitch angle of the long axis of the body was 75.9±3.47° (N = 4 birds) below the horizontal. In this orientation the ducks paddled at an average frequency of 3.5±0.2 Hz. At the transition from the recovery phase to the power phase the collapsed webbed area of the foot was spread abruptly by adduction and dorsiflexion of the digits, as described previously for ducklings swimming on the water surface [10]. At the end of the power phase the transition to the recovery phase was more gradual taking the last 15% of the paddling cycle. In the transition to the recovery phase, the foot moved medially before abduction and plantarflexion of the digits. There was little motion of the foot backwards or dorsally during the transition. On average, the recovery phase lasted 37.3±3.0% of the paddling cycle while the power phase, including the transition to the next recovery, lasted the remaining 62.7% of the paddling cycle duration.

**Figure 3:**
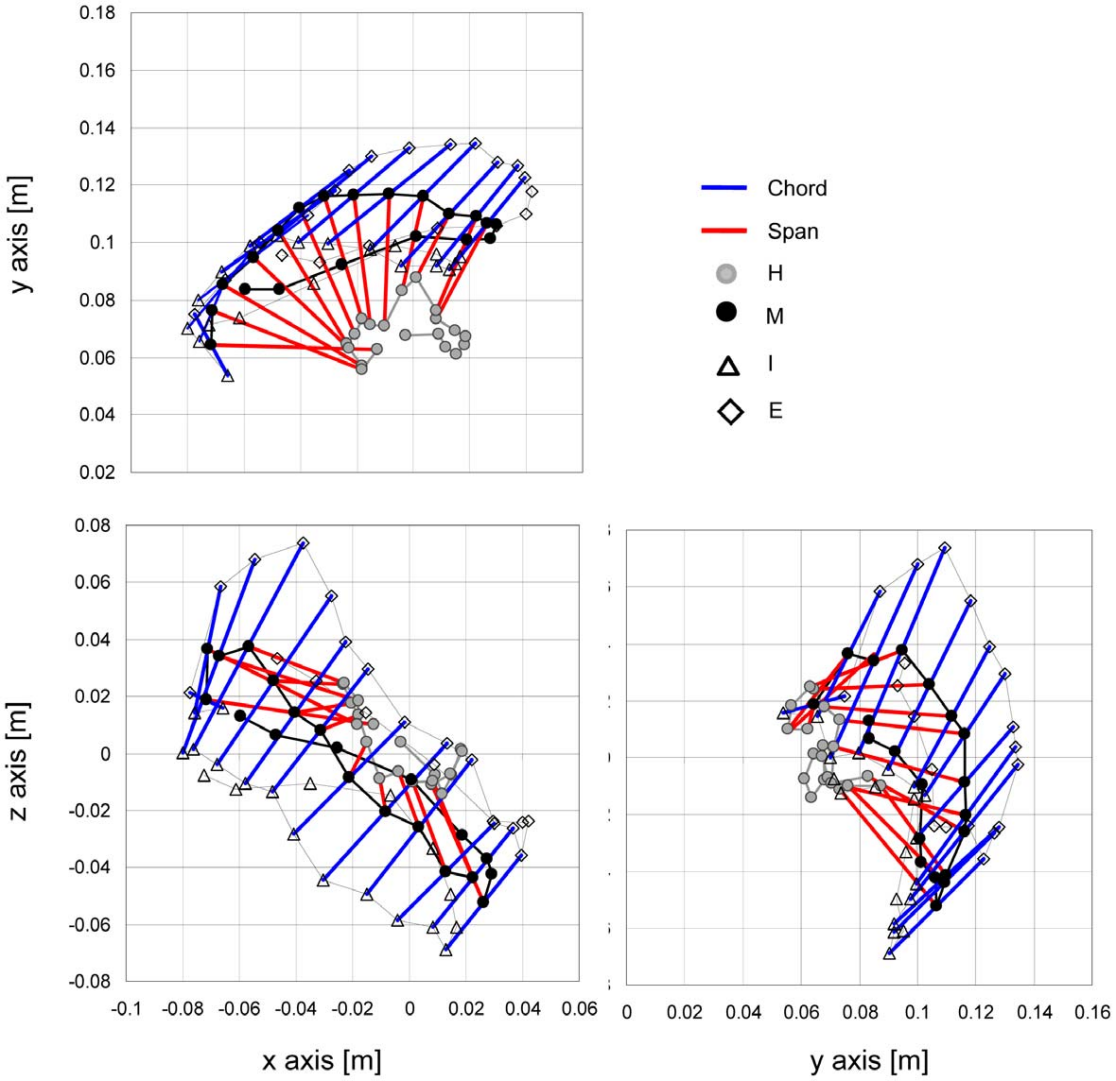
The 3D kinematics of the foot in the duck (x,y,z) frame of reference. The data shown was extracted from two video cameras showing the motion of the left foot of a duck during a single paddling cycle. Shown are the positions of the tip of the internal (I, see Fig. 1) and external (E) toes as well as the calculated tip of the mid toe (M) and the base of the foot (H) every 5% of the paddling cycle duration. The blue lines connecting E and I represent the chord of the foot, the red lines are the span (HM in Figure 1) of the foot. These lines are shown only for the last 60% of the paddling cycle when the webbed area of the foot is spread (the power phase). Upper panel shows the motion of the foot in the xy plane, left and right figures in the lower panel show the same motion in the xz and zy planes respectively.

Figure 3 illustrates an example of the kinematics of the left foot, in the duck coordinate system, during one paddling cycle. During the power phase, in the xy (coronal) plane the span of the foot (HM in Fig. 1) translated backwards relative to the duck as well as rotating by approximately 90°. The foot also moved laterally away from and then towards the midline of the body. The projection of the chord (IE in Fig. 1) on the xy plane was kept in a fairly fixed orientation. In the xz (sagittal) plane the foot moved mostly backwards and up (dorsally) during the power phase. The chord was practically perpendicular to the trajectory of the foot. The stroke plane was inclined relative to the coronal plane of the body by ∼40° (Fig. 3), so given that the average pitch angle of the duck during this cycle was 76°, the trajectory of the foot had a large vertical component in the earth coordinates. In the yz (transverse) plane, the most conspicuous feature was that the foot moved closer to the body along the y (lateral) axis during the recovery when the digits were abducted.

**Figure 4:**
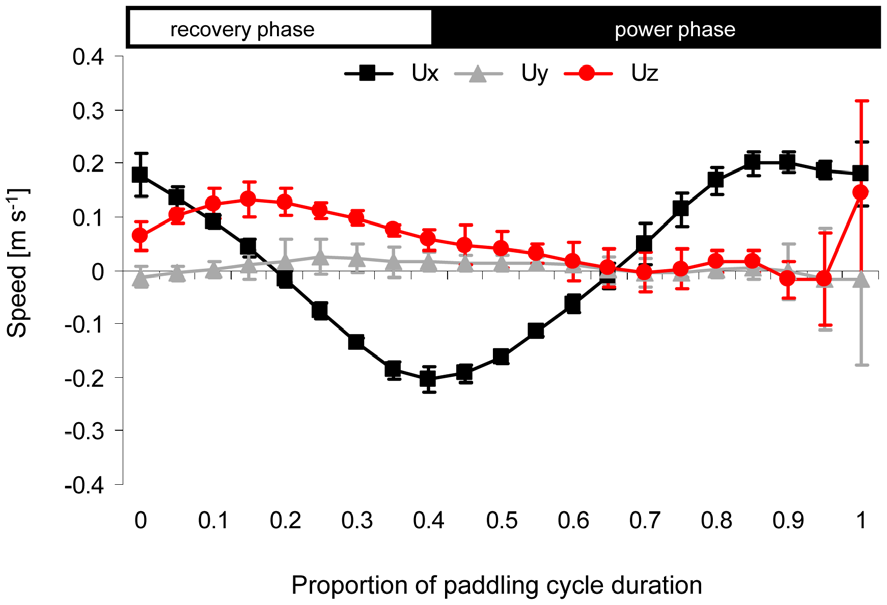
Velocity of the body in the duck frame of reference. The average velocity of the body shown as components along the three axes fixed on the duck. Ux, Uy and Uz are the component of velocity along the long, lateral and dorsoventral axes of the body respectively (see Fig. 2 and Appendix S2). The time (horizontal) axis is normalized time within a paddling cycle. The horizontal white and black rectangles at the top illustrate the division of the paddling cycle into the power and recovery phases. Data averaged from N = 4 birds, ± SD.

The body did not remain stationary during the paddling cycle, moving up and down. In the duck's frame of reference, curves depicting the change in velocity of the body (**U_b_**) along the x axis with time (Fig. 4), showed an acceleration backwards (negative) during the recovery phase and acceleration forward (positive) during the power phase. However, the speed of the duck along the x axis lagged behind the movement of the foot so that at the beginning of the recovery phase the body was moving forward before stopping at ∼20% through the paddling cycle. Subsequently, the body started accelerating backwards. At 40% of the paddling cycle, when the transition between recovery and power phases occurred, the backwards motion of the body was at maximum speed before starting to decelerate. The body stopped and commenced moving forward half way into the power phase (at about 65% of cycle duration). The body also had a positive component of velocity along the z axis (dorsally) during the first 60% of the paddling cycle. Given the large pitch angle of the body this velocity component resulted from the vertical motion of the body during the recovery phase when the duck was floating up in the feeding posture. Since we specifically selected sequences with little yaw or lateral motion of the duck, it was not surprising that the average lateral speed of the duck was negligible compared with movements in the x and z axes.

**Figure 5:**
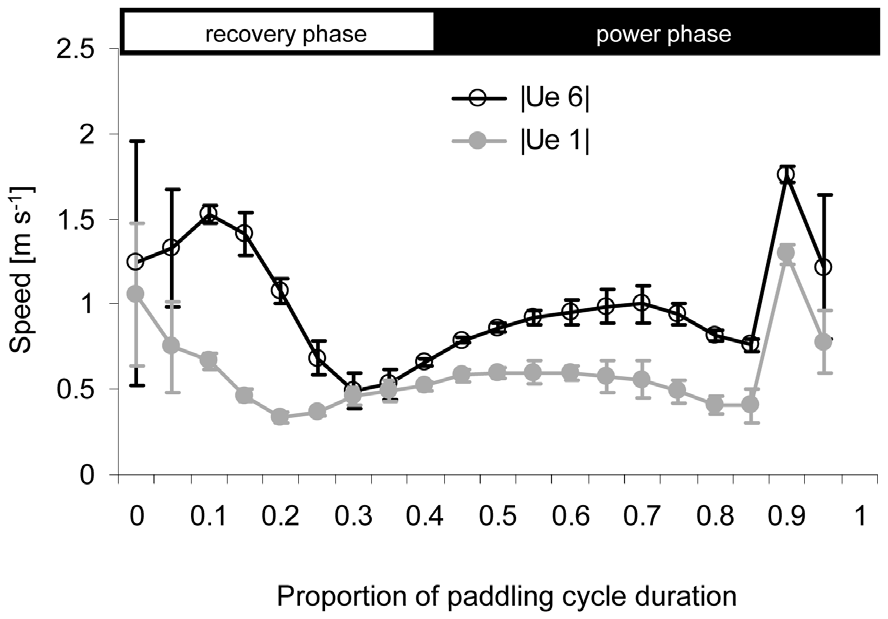
Speed of the foot relative to water. Shown are the root-mean-square speed of the proximal (e1) and distal (e6) foot elements during the paddling cycle. The horizontal axis is the same as in Fig. 4. The horizontal white and black rectangles at the top illustrate the division of the paddling cycle into the power and recovery phases. Data averaged from N = 4 birds, ± SD.

Due to the phase shift between velocity of the body and velocity of the foot along the x axis, the body and foot moved in the same direction during the first half of the recovery and power phases whereas the body and foot moved in the opposite direction in the second half of each phase. As a result motion of the body increased the speed of the foot relative to water during the start of each phase and decreased it at the end so that, during a stroke, variability of the speed of the foot relative to water (**U_r_**) was reduced. Figure 5 shows the root mean square of speed relative to water calculated for foot element 1 (most proximal) and 6 (most distal). Neither element reaches zero speed at the end of each phase (0.4 and 1.0 of the cycle duration) due to the movement of the body. The relative velocity of the distal element was almost 2-fold higher than the velocity of the proximal section, in some parts of the paddling cycle.

**Figure 6:**
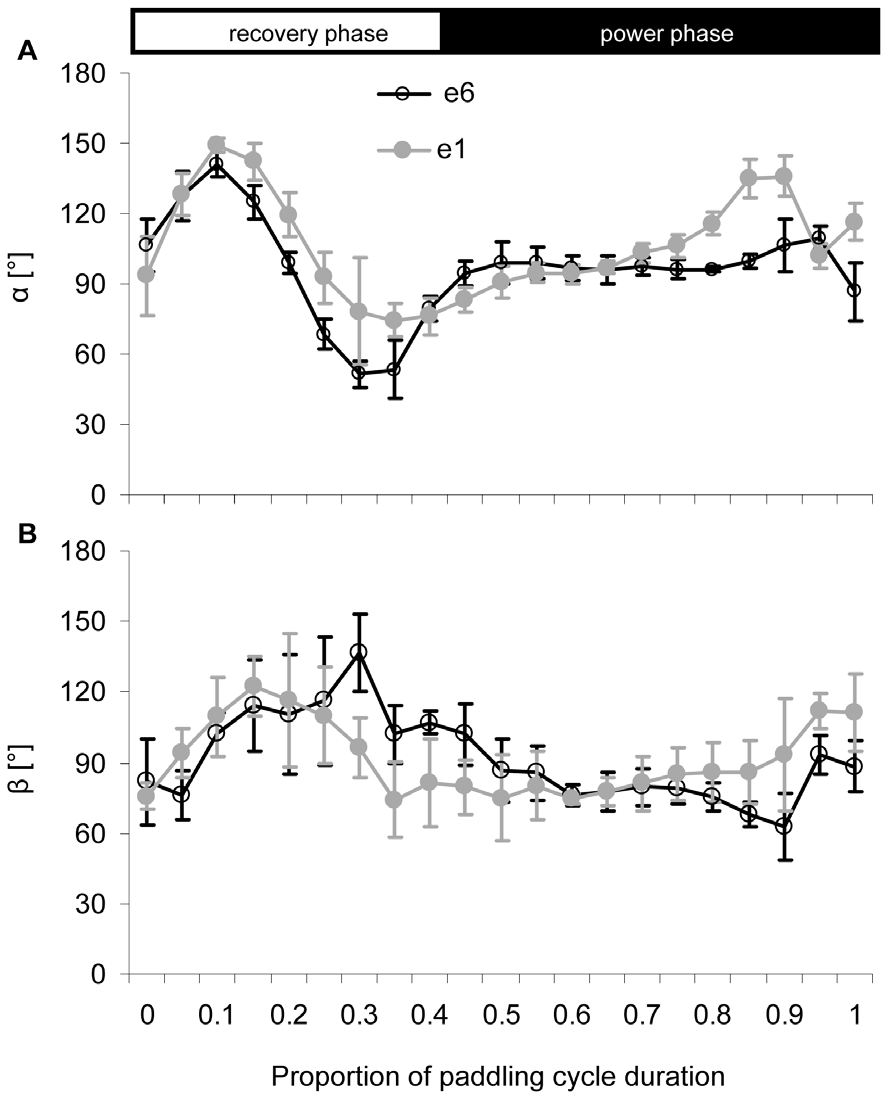
Orientation of the foot during paddling. The angles α (A) and β (B) between the span and chord of the foot respectively and the local relative velocity vector of the foot sections (See Figure 1 for identification of the angles and their notation). Only the angles for foot sections 1 (grey) and 6 (black) are shown. The horizontal axis is the same as in Fig. 4. The horizontal white and black rectangles at the top illustrate the division of the paddling cycle into the power and recovery phases. Data averaged from N = 4 birds, ± SD.

**Figure 7:**
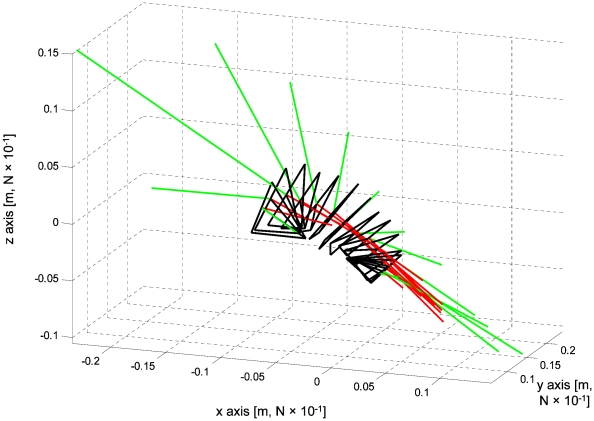
Contribution of drag and acceleration-reaction to the propulsive force. Shown is a 3D representation of the kinematics (in the duck's frame of reference) of the left foot during the power phase of an average paddling cycle (averaged from all birds, N = 4). Each triangle illustrates the position and orientation of the foot separated at 5% time intervals of the total paddling cycle duration. Added to each foot position are scaled vectors showing the mean normal force (red) and mean acceleration reaction (green) generated by the entire foot for that time step (averaged from the 4 birds). The magnitude of the vectors in the graph are reduced by a factor of 10 to fit on the same scale grid as that of the foot kinematics (i.e., in the figure the grid for foot kinematics is in meters and for forces it is in Newton x10^−1^).

**Figure 8:**
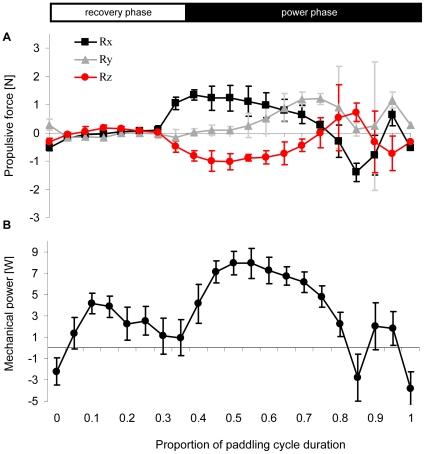
Hydrodynamic force and power of paddling. A) The mean x, y, z components (see Fig. 2) of the resultant propulsive force (R) for the entire left foot as estimated by the model from the foot kinematics (N = 4± SD). Positive values refer to forward (x), left (y) and dorsal (z) in the bird's frame of reference. B) The instantaneous mechanical power exerted for moving both feet through water during the paddling cycle (twice the power calculated for only the left foot) (N = 4± SD). The horizontal axis is the same as in Fig. 4. The horizontal white and black rectangles at the top illustrate the division of the paddling cycle into the power and recovery phases.

Figure 6 shows the mean orientation angles (α and β) of the same foot elements relative to local velocity vectors. During the power phase the foot was oriented perpendicular to the direction of motion in both the angle between the span and velocity (α) and the angle between the chord and velocity (β) and thus, functioned as a drag producing paddle, not a lift producing hydrofoil. Figure 7 uses kinematic data averaged from all birds to show the calculated direction and magnitude of the normal force and acceleration reaction vectors in the xyz (duck) coordinate system during the power phase (last 65% of the paddling cycle). Acceleration reaction is the force resulting from accelerating both the mass of the foot and the mass of water displaced by the foot (see Appendix S1). This force dominates the start and end of the power phase but the force has different directions. Acceleration reaction adds to the propulsive force at the start of the power phase but reduces it at the end of the phase. Figure 8 shows the time distribution of the total propulsive force produced by one foot over the paddling cycle duration. The forces produced during the recovery were minor due to the reduced area of the foot while the power phase provided propulsive force both forward (Rx) and ventrally (Rz) relative to the duck. The stroke also generated lateral force (Ry) in the second half of the power phase. The direction of this lateral force was away from the bird (i.e. to the left of the bird for the left foot). Most of the propulsive force was generated between 0.35 to 0.75 of the paddling cycle duration whereas in the transition to the next recovery some of the propulsive force was reversed due to the acceleration reaction of the foot (Fig. 7). At the beginning of the power phase (between 40%–80% of the cycle), the resultant force in the xz (saggital) plane of the bird was directed at 25.5±1.87° ventral to the long axis (x) of the bird. The force created in this plane by both feet during this time period was 2.7±0.59 N. With the bird pitched at 76°, 98% of this force was directed vertically in the earth coordinate system (see figure 2B), thus working directly against buoyancy. Figure 8B shows the instantaneous mechanical power required for moving both feet through water. The integration of the power requirement over the entire paddling cycle duration gave the energy (work) expended in a paddling cycle as 1.1±0.17 J cycle^−1^. Multiplying work by the paddling frequency gave an average mechanical power to hold position near the 1.5 m deep feeding tray of 3.7±0.47 W.

## Discussion

While feeding at 1.5 m depth, Barrow's goldeneye ducks maintain their vertical position in the water column against buoyancy by pitching the body at 76° from the horizontal and paddling continuously. Most of the propulsive force generated during the power phase was directed on average 25.5° below the bird so that neglecting the lateral component of the propulsive force, 98% of the propulsive force projected on the sagittal (xz) plane was directed vertically to resist buoyancy. Ducks use foot propulsion to achieve a force balance with buoyancy to hold position near the bottom while feeding because the lateral components of the propulsive force are cancelled out by the synchronous paddling of the left and right foot.

During the power phase the webbed area of the foot was kept almost perpendicular to the direction of motion of the foot relative to the water. The major forces involved in propulsion are, therefore, drag and acceleration reaction along the line of motion of the foot (i.e. in the same direction or opposite to it). A growing body of evidence suggests that some foot propelled waterbirds use hydrodynamic lift for propulsion during horizontal swimming where the foot is moved at small angles of attack relative to the water and lift is produced perpendicular to the motion of the foot [14], [17]–[18]. In the special case of holding position near the bottom, body speed is low contributing little to the velocity of either foot relative to water. Furthermore, the motion of the body is largely vertical, which is the same as the motion of the feet so a large change in the direction of relative velocity of the foot is not expected. In contrast, fast horizontal swimming with the body pitched towards the bottom adds a horizontal component to the velocity of the feet relative to the body. This reduces the angle of attack of the webbed foot and changes the relative motion of the foot from a horizontal to vertical trajectory allowing a shift to lift-based swimming. Currently, there is insufficient data on the kinematics of foot propulsion in horizontally swimming ducks to verify whether ducks can also use lift based propulsion. In contrast to piscivorous birds, however, many ducks feed on sessile, benthic food and therefore dive vertically to the bottom and back. For this type of foraging behavior specialization for maintaining position near the bottom may be more advantageous than adaptation for lift based swimming at fast horizontal speeds.

Drag-based swimming is often divided into rowing or paddling based on the plane of motion of the paddle. It is customary to use the term rowing for paddle motion in the horizontal plane and paddling for motion in the vertical plane [23]. When swimming at the surface ducks paddle in the water, while figure 3 shows that when station holding underwater, the stroke is mostly confined to a plane that is inclined by 30–40° to the coronal (xy) plane and involves both lateral and dorsal motion. Thus the motion of the feet is intermediate between paddling and rowing. Qualitative observations showed that the tibia of ducks has freedom to rotate the axis of the tibiotarsal-metatarsal joint so that the arc formed by the feet can be changed from a vertical to a horizontal plane. As a result the feet can be moved on both sides of the body rather than directly below it. Rotating the arc of the foot from a sagittal to a more coronal plane is probably useful for stability and control of orientation while holding position. By rotating and spreading the legs laterally ducks minimize the large pitching moments that would arise from paddling ventrally to the body [24]. Paddling ventrally results in a strong head-up pitching moment during the power phase making it harder for the ducks to move their feet vertically. The lateral position of the foot replaces some of the pitching moment with a lateral moment that is cancelled by both the left and right feet stroking at the same time. Furthermore, the lateral moments are useful since voluntary asymmetries between the motion of the left and right feet can be used to rotate the body (yaw) during feeding, without changing the vertical component of thrust used against buoyancy.

The vertical force (in the earth coordinate system) of the average power phase produced by both feet at 40–80% of the paddling cycle was found to be 2.7 N. This force is used to counter buoyancy. The body mass of the 4 ducks used in the study was 0.670±0.125 kg. The buoyancy of Barrow's goldeneye ducks is 4.25 N kg^−1^ according to a previous study [7] yielding an average buoyancy for our ducks of 2.85 N. Reduction of buoyancy with dive depth in the similarly sized (and similarly buoyant) Lesser scaup is estimated as 15% at 1.2 m depth and 25.5% at 2 m depth [25]. Thus buoyancy of our ducks at 1.5 m depth should be ∼19% lower, or 2.31 N. Therefore, birds move down during the power phase but during the remainder of the paddling cycle the forces generated are too low and the body moves up due to buoyancy.

Our model estimates the propulsive force by assuming that the hydrodynamic function of the foot resembles that of a low aspect ratio plate in a quasi-steady flow. The model appears to predict both the direction and magnitude of the propulsive force during the first 80% of the paddling cycle in agreement with the observed motion of the body. Unfortunately, the model does not adequately predict forces associated with the transition from the power to the next recovery phase. At the transition the model predicts that the forces in the xz plane are reversed, working with buoyancy instead of against it. This is due to the large effect of foot inertia at the end of the stroke. As the foot is brought to a stop, inertia will be in the same direction as the motion of the foot (i.e. up). However it is almost certain that steady flow phenomena will not predominate during this transition. The disparity between the calculated forces and observed kinematics may be the result of unsteady flow effects associated with vortex shedding at the transition that are not adequately represented by the model.

According to our model the average mechanical power exerted by the two feet during station holding was 3.7±0.47 W, or mass specifically 5.5 W kg^−1^. Two previous studies on mechanical power in diving ducks reported power output for staying at the bottom about half of our value (2.51, 2.54, 1.69 W kg^−1^ for Canvasback, Redhead and Lesser scaup respectively [8], and 2.55 W kg^−1^ for Lesser scaup [9]). The large difference for power reported in the present analysis compared with previous studies stems from the fact that we report total mechanical power exerted in paddling whereas the earlier values refer only to the power output (i.e. the power needed to move the body). Power output will be the same as total power only when the feet are 100% efficient in converting all the momentum from the water moved into thrust that is directed in the direction of swimming. Since some of the energy spent on producing the propulsive force is always lost during swimming, power output is always lower than total power and the ratio between the two is the propulsive efficiency. For drag-based swimming, estimates of propulsive efficiency vary from 16% to 33% [26]–[27].

In eco-physiological modeling of diving energetics it is often useful to convert mechanical energy spent during a dive to a more biological currency of energy such as metabolic rate (often measured as the amount of oxygen consumed by the duck). The total mechanical power spent holding position near the bottom should be considerably lower than the metabolic power to account for the energy lost converting chemical energy to mechanical energy (i.e. ‘aerobic efficiency’, see [28] and below). To verify this we estimated the metabolic rate of diving in our ducks based on data from the literature. The allometry of resting metabolic rate (RMR) on body mass in diving ducks (RMR = 446 m^0.98^ where m = mass is in kg and the RMR is in kJ day^−1^) [29] predicts a metabolic rate at rest of 3.49 W for ducks of the mean body mass of our ducks. This value is for ducks resting in air at their thermoneutral zone. Ducks resting on water have a metabolic rate which is at least 1.4 fold higher even when water temperature is the same as the air [30]. The metabolic rate of similarly sized tufted ducks (0.6 kg) diving to 1.5 m was 3.5 times their metabolic rate resting on water [31]. According to these values the metabolic cost of diving for our ducks should be 17.1 W (RMR from the allometric equation x 1.4 for resting on water x 3.5 for diving to 1.5 m). This value is for a dive to 1.5 m that includes not only the energy spent feeding at the bottom but also during a short commute from the surface to the bottom and back. Mechanical energy expenditure is highest when descending to the bottom against buoyancy and lowest (zero) during the passive ascent to the surface which is driven by buoyancy. Nevertheless, we note that the 3.7 W found by our model for mechanical power required to stay near the bottom, at 1.5 m depth, is roughly 21% of the predicted metabolic rate during a dive to that depth. In other words, although our value for mechanical power for staying at the bottom is considerably higher than previous estimates, it is still low enough to account for less than a quarter of the metabolic rate of the dive, leaving ample room for the extra energy spent descending through the water column and losses associated with the conversion of metabolic energy into mechanical energy.

These losses are reflected in the exercise physiology literature as ‘aerobic efficiency’ and can be calculated from the ratio of mechanical work performed to measured oxygen consumed (a measure of metabolic rate) [28]. When aerobic efficiency is known it can be used for converting the estimate of mechanical work done during the dive to the more ecologically relevant currency of energetic needs, i.e. metabolic rate. Both power output (used in the previous models) and propulsion power (estimated here) can be converted to metabolic energy. However, the values of aerobic efficiency used for the conversion in the two cases would be different. For the same dive, the 2 fold difference between the power spent on propulsion for staying near the bottom (reported here), and the power output (reported previously [8]–[9]) would result in a calculated aerobic efficiency (specific for bottom feeding) that is 2 fold larger in our case. This is because the aerobic efficiency calculated from power output subsumes the propulsive efficiency making the conversion from metabolic to mechanical work seem less efficient. Hence, for swimming, power output and power spent on propulsion are different currencies of the mechanical work of swimming. Both can be used for estimating metabolic energy for ecological modeling, using different values of aerobic efficiency.

However, the major advantage in our modeling approach becomes clear in the special case of holding position. When the body is stationary the work done on it is zero since by definition work is the product of the force applied and distance moved. To overcome this problem, previous studies used indirect estimates of work for station holding based on an estimated distance that the body would have moved had the duck stopped paddling. The advantage of the analysis presented here is that we can estimate the work of holding position regardless of whether the body is moving or not, by using the kinematics of the feet. Furthermore, in addition to energy expenditure, our approach provides information on the source of the propulsive force, its magnitude and direction, and the various mechanical factors that drive energetic costs.

The advantages of the current approach are not limited to the special case of holding position near the bottom. Although the values reported here for propulsive force and power are specific to ducks holding position at a depth of 1.5 m, the same blade-element model described here can be applied when birds are swimming horizontally, vertically (resulting in a decrease in buoyancy with depth), holding position near the bottom or engaging in complex maneuvers. Thus the model described here can prove a powerful tool for estimating energy expenditure for complex, real-life foraging behaviors of diving birds; enhancing our understanding of the eco-physiology of these remarkable divers.

## Supporting Information

Appendix S1 Blade-element model for estimating the propulsive force.
(0.22 MB DOC)

Appendix S2 Axes and transformation of the coordinate system.
(0.18 MB DOC)

## References

[1] Heath JP, Gilchrist HG, Ydenberg RC (2006). Regulation of stroke pattern and swim speed across a range of current velocities: diving by common eiders wintering in polynyas in the Canadian Arctic.. J Exp Biol.

[2] Richman SE, Lovvorn JR (2008). Cost of diving by wing and foot propulsion in a sea duck, the white-winged scoter.. J Comp Phys B.

[3] Lovvorn JR, Grebmeier JM, Cooper LW, Bump JK, Richman SE (2009). Modeling marine protected areas for threatened eiders in a climatically changing.. Bering Sea Ecol Applic.

[4] Wilson RP, Hustler K, Ryan PG, Burger AE, Noldeke EC (1992). Diving birds in cold water: do Archimedes and Boyle determine energetic costs?. Am Nat.

[5] Lovvorn JR, Jones DR (1994). Biomechanical conflicts between adaptation for diving and aerial flight in estuarine birds.. Estuaries.

[6] Kortright FH (1962). The ducks Geese, and swans of north America..

[7] Lovvorn JR, Jones DR (1991a). Body mass, volume, and buoyancy of some aquatic birds, and their relation to locomotor strategies.. Can J Zool.

[8] Lovvorn JR, Jones DR, Blake RW (1991). Mechanics of underwater locomotion in diving ducks: drag, buoyancy and acceleration in a size gradient of species.. J Exp Biol.

[9] Stephenson R (1994). Diving energetics in Lesser Scaup (*Aythyta affinis*, Eyton).. J Exp Biol.

[10] Aigeldinger T, Fish FE (1995). Hydroplaning by ducklings: overcoming limitations to swimming at the water surface.. J Exp Biol.

[11] McPhail LT, Jones DR (1998). The relationship between power output and heart rate in ducks diving voluntarily.. Comp Biochem Physiol A.

[12] Baudinette RV, Gill P (1985). The energetics of flying and paddling in water. Locomotion in penguins and ducks.. J Comp Physiol B.

[13] Walker JA, Westneat MW (2000). Mechanical performance of aquatic rowing and flying.. Proc R Soc Lond B.

[14] Johansson CL, Norberg AR (2003). Delta wing function of webbed feet gives hydrodynamic lift for swimming propulsion in birds.. Nature.

[15] Fish FE (1995). Kinematics of ducklings swimming in formation: consequences of position.. J Exp Zool.

[16] Woakes AJ, Butler PJ (1983). Swimming and diving in tufted ducks, *Aythya fuligula*, with particular reference to heart rate and gas exchange.. J Exp Biol.

[17] Johansson CL, Norberg UM (2001). Lift based paddling in diving grebe.. J Exp Biol.

[18] Ribak G, Weihs D, Arad Z (2004). How do cormorants counter buoyancy?. J Exp Biol.

[19] Blake RW (1979). The mechanism of labriform locomotion I. Labriform locomotion in the angel fish (Pterophyllum eimekei): an analysis of the power stroke.. J Exp Biol.

[20] Gal JM, Blake RW (1988). Biomechanics of frog swimming II. Mechanics of the limb beat cycle in *Hymenochirus bottgeri*.. J Exp Biol.

[21] Ellington CP (1984). The aerodynamics of hovering insect flight III. Kinematics.. Phil Trans R Soc Lond B.

[22] Hedrick TL (2008). Software techniques for two- and three-dimensional kinematic measurements of biological and biomimetic systems.. Bioinsp Biomim.

[23] Vogel S (1994). Life in moving fluids..

[24] Ribak G, Weihs D, Arad Z (2008). Consequences of buoyancy to the maneuvering capabilities of a foot-propelled aquatic predator, the great cormorant (*phalacrocorax carbo sinensis*).. J Exp Biol.

[25] Lovvorn JR, Jones DR (1991b). Effect of body size, body fat, and change in pressure with depth on buoyancy and costs of diving ducks (*Aythya spp*.). Can J Zool.

[26] Blake RW (1980). The mechanics of labriform locomotion II. An analysis of the recovery stroke and the overall fin-beat cycle propulsive efficiency in the angelfish.. J Exp Biol.

[27] Fish FE (1996). Transition from drag-based to lift based propulsion in mammalian swimming.. Am Zool.

[28] Lovvorn JR (2007). Thermal substitution and aerobic efficiency: measuring and predicting effects of heat balance on endotherm diving energetics.. Phil Trans R Soc.

[29] Miller MR, Eadie JM (2006). The allometric relationship between resting metabolic rate and body mass in wild waterfowl (Anatidae)and an application to estimation of winter habitat requirements.. The Condor.

[30] Prange HD, Schmidt-Nielsen K (1970). The metabolic cost of swimming in ducks.. J Exp Biol.

[31] Butler PJ (2004). Metabolic regulation in diving birds and mammals.. Respiratory Physiology & Neurobiology.

