# Supplementary material for: Drag-Based ‘Hovering’ in Ducks: The Hydrodynamics and Energetic Cost of Bottom Feeding
Source: PLoS One. 2010 Sep 7;5(9):e12565. doi: 10.1371/journal.pone.0012565 (PMC2935360; doi:10.1371/journal.pone.0012565)
Supplement: Appendix S1 — Blade-element model for estimating the propulsive force. (0.22 MB DOC) [file pone.0012565.s001.doc]

S1. APPENDIX A. - Blade-element model for estimating the propulsive force.

Figure 1 of the main text illustrates the modeling problem and the notation used in the analysis. The area of the webbed foot (HIE in Fig 1) is confined by digits 1 and 3 (HI and HE where H is the proximal vertex, the joint connecting the toes with the tarsus-meta-tarsus). For simplicity we assumed this area to be a flat (confined in a 2-dimensionsional plane) equilateral triangle with digit 2 (HM) the angle bisector of the apex (H) of this triangle, so that point M was half way between I and E. This simplified geometry reduced the number of parameters in the calculations while yielding a reasonably true representation of the shape of the foot. During propulsion the leg bones rotated about the joints in a combined action that resulted in a curved trajectory of the foot. The angular component of this motion implies that the tangential speed of a point along HM increased with distance from the center of the curved trajectory (from H to M). As a result, the contribution of the more distal sections of the foot to the propulsive force was expected to be higher than proximal sections, a phenomenon further enhanced by the increased area of the foot towards the distal edge. Additionally, the velocity of the foot relative to water *(***U_r_**) is a combination (vector summation) of the velocity of the foot relative to the body (**U_f_**) and velocity of the body (**U_b_**).

Eq A1. **U_r_** = **U_b_** + **U_f_**

The magnitude of **U_r_** depended on the position along HM because **U_f_** changed along HM while **U_b_** did not. When **U_b_** and **U_f_** are not collinear, the direction of **U_r_** varied along HM resulting in a change of the local geometric angle of attack. The “blade-element” approach was used to account for changes along HM.

Gal and Blake [A1] described a 2-dimensional blade-element analysis to study propulsion in frogs. The foot was divided into sections along the span, and the hydrodynamic forces were estimated from the velocity of each section relative to the water and the resulting angle-of-attack. The forces calculated for the different sections were then integrated along the span of the foot to yield the force for the entire foot. We used a similar modeling approach for 3-dimensional analysis of the foot of a duck. The foot was divided into N=6 sections with equal width along HM (width=HM/6). By our definition, HM is a symmetry line that defines one axis of the foot area. We term this axis ‘span’ to comply with the terms used in blade-element models. The local chords of the foot were defined as lines in the plane of the foot that were perpendicular to HM. The angle of the apex with the digits fully abducted was taken as ε = 60° based on measurements made on images of the feet of 6 ducks (average ± SD angle = 63° ± 5.5°, n= 6). The length of HM in these images was 6.5 ± 0.2 cm. With the digits fully adducted to collapse the webbed area of the foot, the width (chord) of the foot reduced to the combined width of the two external toes, which was less than 1 cm wide. We took the angle of the apex during the recovery to be ε =10°. From the apex angle and triangular shape, the aspect-ratio (ratio of the span to the average chord, = span^2^/area = (tan ε/2)^-1^) of the foot during the stroke was 1.73.

Therefore in estimating the propulsive force we used results taken from wind tunnel studies on low-aspect ratio plates with free edges [A2]. When plates are tilted at a large angle (γ >45°) to the oncoming flow, the pressure difference between the two sides of the plate results in a hydrodynamic force that is directed perpendicularly to the plate [A2]. The axial force components (parallel to the plate) result from skin friction and are much smaller and were neglected [A3]. The normal force (*F_N_*) generated can be described in the form:

Eq.A2 $F_{N}=\frac{1}{2}\rho AC_{N}U^{2}$

where ρ is water density (taken as 1000 kg m^-3^), *A* is the area of the plate, *U* is the speed of the plate relative to water (here *U_r_*), and *C_N_* is the normal force coefficient. The direction of *F_N_* is normal to the plate on the side opposing the direction of motion. As pointed out by Blake [A3], wind tunnel experiments show that the force normal to a low aspect-ratio plate with free edges remains fairly constant when the plate is oriented in respect to the flow through a range of angles γ =90 ± 45° where γ is the angle of attack. Hence, *C_N_* in Eq. A2 can be assumed to be constant as long as the orientation of the foot relative to its direction of motion is close to perpendicular. For plates with an aspect-ratio between 0.5 - 5.0 the value of *C_N_* is constant at *C_N_*=1.17 [A2]. For the case of γ < 45°, wind tunnel studies show that C_N_ = 0 when γ=0 and increases with sin γ to a maximum value of 1.8 at ~40-45° [A2]. For such cases in our model we adopted the solution suggested by Blake [A3]:

Eq. A3 *C_N_  =* 1.17 (for 45° < *γ* <135°)

*C_N_ =* 2.5 *sin γ* (for *γ*<45° or *γ* >135°)

and used the appropriate *C_N_* in Eq. A2.

In a previous analysis Gal and Blake [A1] described a method to calculate the two dimensional velocity of foot sections based on radial distances and angular positions of the various leg joints taken from cine’ films. This was not possible in our case because not all leg joints are visible and the motion of leg segments is not necessarily limited to a single plane of the video image. Therefore the speed of each foot section was calculated numerically using a four-point parabola [A4] from change in position of the center of hydrodynamic force with time. This center was assumed to be located on HM at 0.6 of the distance between the proximal and the distal borders of each section. This assumption stems from the trapezoid shape of the elements where the radius of the second moment of area of the elements converges to 0.6 at the distal edge of the foot. The time derivative of the 3D positions along HM yields a 3D velocity vector for each foot element (**U_r_**). The orientation of the foot element relative to its **U_r_** vector was described using angles α and β formed by the axes of the foot (Fig. 1). α is the angle between the two vectors **U_r_** and **HM** that define the velocity-span plane (ABCD in Fig 1) in 3D space. The angle β is the angle between the local chord and **U_r_** so that when 0<β<180°, the chord is inclined relative to the ABCD plane. In Fig. 1, β is the dihedral angle formed between the plane ABCD and the plane of the flat foot (HIE). Since **U_r_** is calculated for each foot element, each element has its own instantaneous α, β and **U_r_** at each time step in the paddling cycle. In vector form, the angle α is found from the scalar product of **HM** and **U_r_**.

Eq.A4 $\alpha=\cos^{-1}\left(\frac{\mathbf{U}_{\mathbf{r}}\cdot\mathbf{HM}}{\left|\mathbf{U}_{\mathbf{r}}\right|\left|\mathbf{HM}\right|}\right)$

To determine β we calculated the angle between the vector normal to the foot plane (**n_HIE_**) and the vector normal to the velocity-span plane (**n_ABCD_**). The vector **n_HIE_** is the vector (cross) product of **HI** and **HE**:

Eq. A5 $\mathbf{n}_{\mathbf{HIE}}=\mathbf{HI}\times\mathbf{HE}$

and **n_ABCD_** is the vector product of **U_r_** and **HM**:

Eq. A6 $\mathbf{n}_{\mathbf{ABCD}}=\mathbf{Ur}\times\mathbf{HM}$

The angle β was obtained from the scalar product of the two normal vectors:

Eq. A7 $\beta=\cos^{-1}\left(\frac{\mathbf{n}_{\mathbf{HIE}}\cdot\mathbf{n}_{\mathbf{ABCD}}}{\left|\mathbf{n}_{\mathbf{HIE}}\left|\mathbf{n}_{\mathbf{ABCD}}\right|\right|}\right)$

Given ***U_r_***, α and β of each foot element, the magnitude of *F_N_* for each element was calculated where the direction of this force was collinear with **n_HIE_**. The normal force represents the hydrodynamic force associated with steady motion. For unsteady motion, a further force (acceleration reaction) arises from the contribution of inertia of the foot plus the mass of water accelerated with it (added mass). The acceleration of each foot section was found in the same manner as velocity by taking the time derivative of **U_r_**. The direction of the acceleration reaction force was opposite to the direction of acceleration. The mass of water accelerated by a flat body moving broad-side into the flow is approximately equal to a cylinder with the width of the body as the diameter [A5]. For the foot element we estimated the added mass as the mass of water occupying a volume circumvented by the area of the foot that is projected onto the flow. Since the foot sections resemble a trapezoid the volume of each foot element was calculated as a truncated cone. The volume for the nth (n=1, 2, 3 …N) foot element is:

Eq. A8a $V_{n}=\frac{1}{3}\pi h\sin\alpha\left[\left(C_{n}\sin\beta\right)^{2}+\left(C_{n-1}\sin\beta\right)^{2}+C_{n}C_{n-1}(\sin\beta)^{2}\right]$

with

Eq. A8b $h=\frac{HM}{N}$

Eq. A8c $C_{n}=\frac{nHM\tan\frac{\varepsilon}{2}}{N}$

Eq. A8d $C_{n-1}=\frac{\left(n-1\right)HM\tan\frac{\varepsilon}{2}}{N}$

where n is increased from H to M and α and β are specific to the nth foot element.

The virtual mass (*m_vn_*) of foot section n is:

Eq. A9 $m_{vn}=\rho V_{n}+m_{fn}$

where *m_fn_* is the mass of the foot section n which in the case of thin flat plates moving broad side to the flow is expected to be much smaller than the added mass of water (*ρVn*). This is especially the case for more distal foot sections which are both thinner and wider. Since in this study, we could not measure  *_m_*fn from live birds it was ignored for the calculation of  *_m_*vn . However, the measured total mass of the foot of a frozen carcass (0.0078 kg) was added to the virtual mass calculation of element n=1 which represents the thickest section of the foot. This was done to ensure that the acceleration of the mass of the foot was not completely disregarded in the analysis. The acceleration reaction force of the nth foot element is the product of virtual mass and acceleration:

Eq. A10 $\mathbf{F}_{\mathbf{An}}=-\mathbf{a}_{\mathbf{rn}}m_{vn}$

where the minus sign denotes that the direction of this force is opposite to the direction of the acceleration relative to water.

Once the normal force and the acceleration reaction were found for each foot element at each time step in the paddling cycle the two forces were combined using vector summation as the instantaneous resultant force of the foot element (**R_n_**). The total propulsive force generated by one foot (**R**) was found by integration of the force over the entire foot (summing all elements). The instantaneous mechanical power (*P_n_*) required for moving a foot element through the water, was the projection of the resultant force on the direction of motion of the foot multiplied by the velocity of the foot section.

Eq A11. *P_n_* = **R_n_** ∙ **U_fn_**

Integrating the instantaneous power from all foot elements along the span of the foot yielded the instantaneous power exerted by the entire foot. Integrating the instantaneous power over the duration of the paddling cycle gave the mechanical work (*W*) done by one foot in a paddling cycle:

Eq A12. $W=\int_{t0}^{t1} \sum_{n=1}^{N} R_{n}\cdot U_{fn}$

where t_0_ and t_1_ are the time at the start and end of the paddling cycle respectively.

Multiplying this work by 2 to account for both feet and by the paddling frequency (*f*) gave the average power spent on staying near the bottom ($\bar{P}$)

Eq. A13. $\overline{P}=2Wf$

References for Appendix A

A1. Gal JM, Blake RW (1988) Biomechanics of frog swimming II. Mechanics of the limb beat cycle in *Hymenochirus bottgeri*. J Exp Biol 138: 413-429.

A2. Hoerner SF (1965) Fluid-dynamic drag. Bakersfield: Hoerner Fluid Dynamics.

A3. Blake RW (1979) The mechanism of labriform locomotion I. Labriform locomotion in the angel fish (Pterophyllum eimekei): an analysis of the power stroke. J Exp Biol 82: 255-271

A4. Rayner JMV, Aldridge HDJ (1985) Three-dimensional reconstruction of animal flight paths and the turning flight of microchiropteran bats. J Exp Biol 118:247-265.

A5. Lighthill MJ (1970) Aquatic animal propulsion of high hydromechanical efficiency. J Fluid Mech 44: 265-301.
