# Supplementary material for: Drag-Based ‘Hovering’ in Ducks: The Hydrodynamics and Energetic Cost of Bottom Feeding
Source: PLoS One. 2010 Sep 7;5(9):e12565. doi: 10.1371/journal.pone.0012565 (PMC2935360; doi:10.1371/journal.pone.0012565)
Supplement: Appendix S2 — Axes and transformation of the coordinate system. (0.18 MB DOC) [file pone.0012565.s002.doc]

S2. APPENDIX B – Axes and transformation of the coordinate system.

Each instantaneous 3D position can be described as a position vector from the origin (0,0,0). The properties of vectors are used to transform the data from the earth (XYZ) coordinate system to the duck (xyz) system by first finding the direction cosines of each of the duck axes in the earth frame of reference. The direction cosines are then used to transform all points between the two systems using a transformation matrix.

First, the pitch angle (θ) of the body was calculated from points T (tail) and N (neck) in the XYZ system from the angle between the 3D vectors **TN** and **Z,** where the latter is the vertical axis in the earth system. The angle was calculated from the scalar product of the two vectors:

Eq. B1) $\theta'=\cos^{-1}\left(\frac{\mathbf{TN}\cdot\mathbf{Z}}{\left|\mathbf{TN}\right|\left|\mathbf{Z}\right|}\right)$

where *θ'* is relative to the vertical and is > 90°.We use θ = θ’-90° as the pitch angle measured relative to the horizontal axis.

The y axis in the duck’s frame of reference is defined in the direction of the line connecting the two leg joints J_R_ and J_L_ in the XYZ frame of reference (Fig. 2). The vector form of **y** was found by vector subtraction of the positional vectors of J_L_ and J_R_:

Eq. B2) $\mathbf{y}=\mathbf{J}_{\mathbf{L}}-\mathbf{J}_{\mathbf{R}}$

therefore the duck’s lateral axis was defined with the positive end pointing to the left of the duck. The unit vector of **y** (denoted by a cap**,)** was found by dividing **y** by its magnitude:

Eq. B3) $\hat{\mathbf{y}}=\frac{\mathbf{y}}{\left|\mathbf{y}\right|}$

The XYZ coordinates of a point J_0.5_ half way between J_L_ and J_R_ was calculated and used as the new origin for the xyz duck coordinate system. The change in position of J_0.5_ with time was used to numerically derive the 3D components of the velocity of the duck (**U_b_)**. Then, the coordinate system was moved to the new origin by subtracting the coordinates of J_0.5_ from all other positions.

The direction of a temporary axis was defined as perpendicular to **y** and horizontal in the earth frame of reference (i.e. perpendicular to **Z**). By this definition is obtained from the vector product of  and **Z** i.e.:

Eq. B4)

where is the unit vector of the vertical axes (0,0,1).

Once and  are known, the direction cosine of the third orthogonal axis () is obtained from the vector product of the others according to the right hand rule:

Eq. B5)

The x and z axes were aligned with the morphological axes of the duck by rotating the coordinate system about the y axis by the pitch angle θ. The rotation matrix to calculate the new vector cosines is:

Eq. B6)

The direction cosines of the , and were used in a transformation matrix, to change the position of any point, P, from the earth coordinate system (PX, PY, PZ) to the duck’s coordinate system (Px,Py,Pz). The expansion of the transformation matrix is:

Eq. B7) Px = PXa_1_+PYa_2_+PZa_3_

Py = PXb_1_+PYb_2_+PZb_3_

Pz = PXc_1_+PYc_2_+PZc_3_

where a_1-3_ are the three direction cosines of , b_1-3_ are the direction cosines of and c_1-3_ are the direction cosines of **.** The transformation matrix was recalculated for every time step in the movies (i.e. video field, 60 fields s^-1^). The components of the velocity of the duck **U_b_** were also transformed into the duck frame of reference
